# Dr. Arthur W. Thomas

**Published:** 1912-04

**Authors:** 


Dr. Arthur W. Thomas of Rochester, a graduate of Syracuse, 1895, died February 29, 1912, aged 39. He was an interne at the Rochester City (now General) Hospital in 1895-'96.

In October 1896, Dr. Thomas was married to Dr. Cornelia White, of Syracuse, who was a classmate in the medical school. He leaves his wife; one son, Robert W. Thomas; his father and mother, Mr. and Mrs. Edgar Thomas, of Fabius, N. Y., and two brothers, C. E. Thomas, of Skaneateles and W. H. Thomas, of Syracuse.

